# Association of TLR4-T399I Polymorphism with Chronic Obstructive Pulmonary Disease in Smokers

**DOI:** 10.1155/2009/260286

**Published:** 2010-02-15

**Authors:** Matthaios Speletas, Vassiliki Merentiti, Konstantinos Kostikas, Kyriaki Liadaki, Markos Minas, Konstantinos Gourgoulianis, Anastasios E. Germenis

**Affiliations:** ^1^Department of Immunology and Histocompatibility, University of Thessaly Medical School, 41110 Larissa, Greece; ^2^Respiratory Medicine Department, University of Thessaly Medical School, 41110 Larissa, Greece

## Abstract

Tobacco smoking has been considered the most important risk factor for chronic obstructive pulmonary disease (COPD) development. However, not all smokers develop COPD and other environmental and genetic susceptibility factors underlie disease pathogenesis. Recent studies have indicated that the impairment of TLR signaling might play a crucial role in the development of emphysema. For this purpose we investigated the prevalence and any possible associations of common TLR polymorphisms (*T* *L* *R*2-R753Q, *T* *L* *R*4-D299G, and *T* *L* *R*4-T399I) in a group of 240 heavy smokers (>20 pack years), without overt atherosclerosis disease, of whom 136 had developed COPD and 104 had not. The presence of *T* *L* *R*4-T399I polymorphism was associated with a 2.4-fold increased risk for COPD development (*P* = .044), but not with disease stage or frequency of exacerbations. Considering that infections contribute to COPD and emphysema pathogenesis, our findings possibly indicate that dysfunctional polymorphisms of innate immune genes can affect the development of COPD in smokers. Although this finding warrants further investigation, it highlights the importance of impaired innate immunity towards COPD development.

## 1. Introduction

Emerging evidence suggests that immune cells accumulate in bronchial biopsy specimens in patients with chronic obstructive pulmonary disease (COPD) [1, 2], questioning in this way whether the lung destruction in COPD is immune cell driven. Tobacco smoking has been identified as the most important risk factor of the disease [3, 4], interfering with the innate host defense system by damaging cells through the attack of oxidants, increasing mucus production, reducing mucociliary clearance, and disrupting the epithelial barrier [1]. Considering that less than 20% of smokers develop COPD [3–5], it has been suggested that additional susceptibility factors might be implicated in lung obstruction and emphysema development [2, 6, 7]. 

It is well accepted nowadays that factors favoring infections contribute to COPD development, since air pollution, latent adenoviral infections, and childhood respiratory infections have been implicated in disease pathogenesis, in a proportion of patients [3, 4, 8]. In this context, recent studies have demonstrated that impaired signaling of innate immunity receptors may contribute to COPD and emphysema pathogenesis [9–11]. Amongst these receptors, Toll-like receptor (TLR) 4 seems to play a pivotal role in lung homeostasis by contributing to the defense of endothelial cells against oxidants [9, 10]. Moreover, TLR4-knockout mice (*T* *l* *r*4^ − /−^ mice) spontaneously develop emphysema, associated with an oxidant/antioxidant imbalance, due to increased Nox3 gene expression and elastin degradation [11]. The impairment of TLR signaling in vivo becomes evident through the presence of single nucleotide polymorphisms (SNPs) that have been associated with receptor hypo-responsiveness and susceptibility to bacterial, fungal, and viral infections (reviewed by Schroder and Schumann) [12]. 

Amongst TLRs, TLR-4 recognizes lipopolysaccharides of Gram (-) bacteria, while TLR-2 recognizes endogenous inflammatory mediators in addition to microbial products like lipotheicoic acid of Gram (+) bacteria [12], namely, the pathogens that play a crucial role, at least, in COPD phenotype (frequency and severity of exacerbation attacks) [13]. Thus, based on the abovementioned data, the present study was designed to analyze the prevalence of common *TLR* SNPs, namely, *TLR2*-R753Q, *TLR4*-D299G, and *TLR4*-T399I, in a cohort of smokers with and without COPD, in order to demonstrate their possible contribution to disease pathogenesis and phenotype.

## 2. Materials and Methods

### 2.1. Study Population

One hundred and thirty-four (134) Greek patients with COPD (male/female: 133/1, mean age: 69.4 years, range: 39–88) and 106 Greek smokers without the disease (male/female: 102/4, mean age: 63 years, range: 40–84), who were randomly selected between October 2005 and October 2008, were enrolled in the study. Individuals with a history of severe cardiovascular comorbidities (including coronary artery disease or stroke) were excluded from the study. Additionally, individuals with coexisting asthma or atopy, autoimmune diseases, and cancer were not enrolled in the study.

All participants had a smoking history of more than 20 pack years (pys) and were current smokers or ex-smokers, the latter being defined as having quit smoking for more than one year. All participants, including normal controls, were submitted to physical examination and spirometry and filled in a detailed questionnaire, including the presence of respiratory symptoms (e.g., cough, sputum, and dyspnoea). Dyspnoea severity was estimated according to the Medical Research Council (MRC) scale. 

COPD diagnosis was based on spirometric data showing airway obstruction (forced expiratory volume in one second [FEV_1_]/forced vital capacity [FVC] < 70%) in individuals with a smoking habit >20 pys, according to the Global Initiative for Chronic Obstructive Lung Disease (GOLD) 2007 guidelines [13]. Classification of COPD severity was based on postbronchodilator FEV1, according to GOLD guidelines (Stage I—mild COPD FEV1 ≥ 80.0% predicted; Stage II—moderate COPD 50.0% ≤ FEV1 < 80.0% predicted; Stage III—severe COPD 30.0% ≤ FEV1 < 50.0%; Stage IV—very severe COPD 30.0% ≤ FEV1, or FEV1 < 50.0% predicted with respiratory failure) [13]. Spirometry was carried out with a dry spirometer (Koko Legend, Ferraris Louisville, CO, USA) according to the American Thoracic Society guidelines [14]. Patients exhibiting three or more COPD exacerbations, or two or more exacerbations including one hospitalization due to COPD in the previous year, were considered frequent exacerbators. The evaluation of exacerbation status was based on patient records and was available in 123 out of 134 COPD patients. Clinical and demographic data of the enrolled individuals are presented in Table 1. 

All subjects provided written informed consent. The study was conducted in accordance with the principles of Helsinki declaration and was approved by the Institutional Review Board of the University Hospital of Larissa, Greece.

### 2.2. Molecular Techniques

Genomic DNA was extracted from peripheral blood using the QIAamp DNA Blood Mini Kit (Qiagen), according to manufacturer's instructions. The detection of the *TLR4*-D299G and *TLR4*-T399I polymorphisms was performed by allele-specific PCR followed by restriction fragment length polymorphism (PCR-RFLP) analysis, as described previously [15, 16]. In brief, the forward primers, in both reactions, were modified at the 5′ end, creating restriction enzyme recognition sites (NcoI for the *TLR4*-D299G polymorphism and HinfI for the *TLR4*-T399I), so that if a polymorphism is present, then PCR-RFLP analysis will create digestion fragments, visible on agarose gels [15, 16]. The detection of the *TLR2*-R753Q polymorphism was also performed by PCR-RFLP, since the presence of polymorphism results in the alteration of DNA sequencing, allowing digestion by SfcI [17]. Primers used for the PCR amplification of *TLR2* gene were previously described by Ogus et al. [18]. All PCR and digestion procedures were carried out in the PCR-engine apparatus PTC-200, MJ-Research (Watertown, Massachusetts), while the PCR and digestion products were analyzed in 2% TBE agarose gels.

For the confirmation of PCR-RFLP results, randomly chosen PCR products, positive and negative for the *TLR* polymorphisms, were purified by Qiagen PCR Purification System (Qiagen, UK) and sequenced using an ABI Prism 310 Genetic Analyzer (Applied Biosystems, Foster City, CA) and a Big Dye Terminator DNA sequencing kit (Applied Biosystems).

### 2.3. Statistical Analysis

Chi-square (*χ*2) test along with Yates' correction was used to compare the allele and genotype frequencies between disease and control groups. Fisher's exact test was used when appropriate. The association between COPD and *TLR* polymorphisms, and the other categorical clinicolaboratory variables of interest, was tested using a univariate logistic regression model. Continuous variables were compared using the nonparametric Mann-Whitney *U*-test. An association was expressed as odds ratio (OR) with the corresponding 95% confidence interval (CI). A variable was considered significant when *P*< .05. The above analyses were performed using SPSS (version 10.0, Chicago, IL, USA). At the end, the deviations from Hardy-Weinberg equilibrium were evaluated using the freely available software Arlequin 3.11 (http://cmpg.unibe.ch/software/arlequin3).

## 3. Results

### 3.1. TLR Polymorphisms Analysis in COPD Patients and Normal Smokers

**Figure 1:**
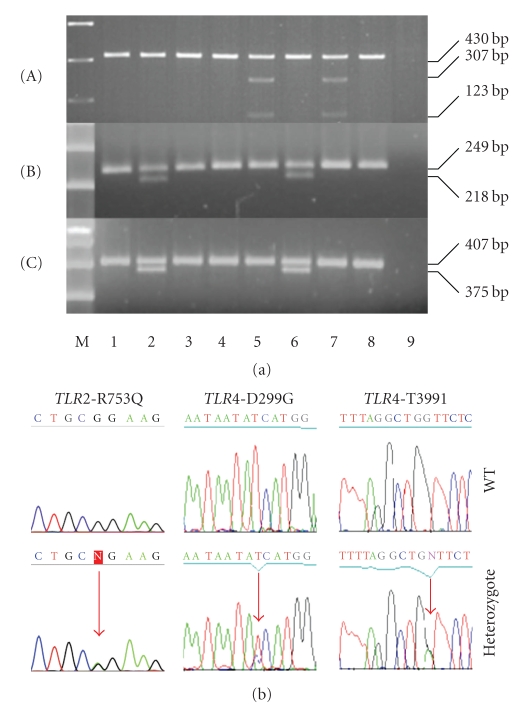
(a) Representative digestions showing the *TLR2*-R753Q (A), *TLR4*-D299G (B), and *TLR4*-T399I (C) polymorphisms. M: 200 bp ladder molecular weight marker. Lanes 1–8: Patients with COPD; lane 9: negative PCR control. Samples without any polymorphism (1,3, 4,8) display undigested PCR products, 430 bp for the *TLR2*-R753Q, 249 bp for the *TLR4*-D299G, and 407 bp for the *TLR4*-T399I. Samples 5 and 7 are heterozygotes for the *TLR2*-R753Q polymorphism, whereas the *TLR2*-753Q allele contains a Sfc I restriction site resulting in 307 bp and 123 bp fragments. Samples 2 and 6 are double heterozygotes for both *TLR4* polymorphisms. In particular, the *TLR4*-299G allele contains an NcoI restriction site resulting in 218 bp and 31 bp fragments, while the *TLR4*-399I allele contains a HinfI restriction site resulting in 375 bp and 28 bp fragments. The digestion products were analyzed on 2% of TBE agarose gels and the 28 bp and 31 bp fragments are not visible on agarose gels. (b) Representative sequencing analysis shows the presence of *TLR2*-R753Q, *TLR4*-D299G, and *TLR4*-T399I polymorphisms.

Examples of the detection of *TLR* polymorphisms by PCR-RFLP are presented in Figure 1. Direct sequencing of 32 randomly chosen samples (5 heterozygotes for both *TLR4* polymorphisms, 2 heterozygotes for the *TLR2*-R753Q, and 25 wild-type) confirmed the results of PCR-RFLP analysis. No one individual was homozygous for any *TLR* polymorphism. The allele and genotype frequencies of the *TLR4*-T399I SNP were significantly increased in patients with COPD when compared to normal smokers (*P* = .046 and *P* = .039, resp.). Moreover, an increased frequency of the *TLR4*-D299G allele in COPD was also observed but failed to reach statistical significance (*P* = .061). Considering the prevalence of the *TLR2*-R753Q, no significant difference between COPD patients and healthy smokers was observed (*P* = .117). Logistic regression analysis revealed that individuals carrying the *TLR4*-T399I polymorphism displayed a 2.4-fold increased risk to develop COPD (95% CI: 1.02–5.64, *P* = .044). Other variables associated with COPD development were age (*P*< .001) and pys (*P*< .001). Considering the impact of the other polymorphisms, no significant difference was observed (Table 2). 

*TLR4*-T399I polymorphism was strongly associated with the value of FEV1/FVC ratio (*P* = .032) but not with disease stage and the frequency of exacerbations (*P* = .130 and *P* = .562, resp.). On the other hand, no significant correlation of the *TLR2*-R753Q and *TLR4*-D299G polymorphisms was observed with the evaluated parameters. 

Moreover, positive strong correlations of COPD stage with pys (*P*< .001) and the frequency of exacerbations (*P*< .001) were found. Finally, the exacerbations of COPD were observed more frequently among heavy smokers (*P* = .015).

### 3.2. The Hardy-Weinberg Principle among TLR SNPs

The analysis suggests that both of the *TLR4* gene polymorphisms obey Hardy-Weinberg principle in the group of COPD patients (*P* = .616 and *P* = .585, for the D299G and T399I SNPs, resp.) and normal smokers (*P* = .064 and *P* = .922, for the D299G and T399I SNPs, resp.). Moreover, *TLR2*-R753Q SNP shares the same deviant behavior in both populations (*P* = .976 and *P* = .940 for COPD patients and normal smokers, resp.). 

To this end, we confirmed that the G allele of *TLR4*-D299G SNP has a high level of linkage disequilibrium with the allele C of *TLR4*-T399I SNP (*P*<; .001), for both COPD patients and normal smokers, as reported previously [19].

## 4. Discussion

To the best of our knowledge, this is the first report examining a possible association of several common *TLR* polymorphisms with COPD development and severity in smokers. Clear evidence is presented for a novel association between *TLR4*-T399I polymorphism and susceptibility to COPD in Greek smokers, in view of the higher frequency of *TLR4*-399I allele in smokers with COPD compared to that of healthy ones. 

We should underscore that the sample size in our study was relatively small, and thus, the power of detecting significant results was limited (almost 20%). However, candidate-gene association studies have the tendency to lack the power to detect a statistically significant association. For example, in order to achieve a power >80% to identify a modest genetic effect (odds ratio 1.2) of a polymorphism present in 10% of individuals, a sample size of 10,000 subjects or more would be needed [20]. Therefore, the sample sizes required to predict association have to be far beyond what is currently available and no single institution or entity alone will be able to provide a reasonable number of patients. However, a future meta-analysis of multiple studies clearly has a role in offering an analysis with the potential for higher power [21]. Future collaborative studies may allow the pooling of data, providing more power to detect significant associations. Furthermore, consortia performing gene-candidate or genome wide associations studies will be able to replicate the validity of the present findings [21, 22].

The two *TLR4* SNPs analyzed in this study (D229G and T399I) are located in the extracellular domain of the human *TLR4.* These result in impaired TLR4 signaling, since they have been associated with receptor hyporesponsiveness in alveolar macrophages and epithelial cells, and peripheral blood mononuclear cells [19]. It has been estimated that the tracheobronchial tree of stable COPD patients is colonized with bacteria and viruses in approximately 30% of cases, many of which are recognized by TLR4, such as *H. influenzae* and *M. catarrhalis *[23]. Thus, any TLR4 dysfunction, such as the presence of T399I polymorphism, might reduce the clearance of the abovementioned pathogens, contributing to COPD pathophysiology. This is in accordance with previous studies where an aberrant innate immune response, being demonstrated by alterations of lung cytokines, oxidant stress, and TLR4 signaling, has been associated with experimental emphysema [7, 9–11]. 

Till now, only two groups have been examined previously, the possible association of the *TLR4*-D299G SNP with COPD development, with conflicting findings [24, 25]. Rohde et al., having included a proportion of nonsmokers in the analyzed COPD group, reported a decreased frequency of *TLR4*-D299G in COPD patients compared to normal controls [24]. However, the interpretation of these results warrants caution, since no smoking habit determination was performed for the control group. For the latter, we consider it to be a very important issue in the design of disease association studies. On the other hand, Sabroe et al. screened a population of 289 smokers and observed no association between *TLR4*-D299G polymorphism and lung function [25]. However, they avoided describing their patients as COPD patients, whilst no details about clinical symptoms defining COPD are given. On the other hand, our findings suggest that there is no association of the *TLR2*-R753Q polymorphism with either the onset or the course of COPD. This is in accordance with the recent work by Pabst et al., where a similar finding was observed analyzing COPD patients and controls, including a significant number of nonsmokers [26]. 

Considering the presence of linkage disequilibrium for the two *TLR4* polymorphisms, we would expect similar results for the *TLR4*-D299G SNP to those observed for the *TLR4*-T399I. However in our study, although the presence of *TLR4*-D299G polymorphism was associated with a 2-fold increased risk for COPD development, this difference did not reach statistical significance (Table 2). It is worth of note that initial in vitro studies indicated that the *TLR4*-D299G genotype might have a greater functional impact compared with that of the *TLR4*-T399I genotype [19]. However, this might not be the case in vivo, since recent gene association studies have shown that the abovementioned SNPs could affect disease development in a different way. For example, Kiechl et al. observed that the *TLR4*-D299G SNP has a more profound protective effect to atherosclerosis risk than *TLR4*-T399I [27], while Török et al. described that only *TLR4*-T399I SNP affects the susceptibility to ulcerative colitis [28]. It is obvious that further studies using appropriate animal models could verify the role of *TLR4*-T399I polymorphism.

To this end, the present study has two major distinguishing factors compared to previous ones. First was the analysis of the *TLR4*-T399I polymorphism and second was the fact that no individuals with overt coronary artery disease or stroke were enrolled, considering the possible contribution of *TLR4* SNPs in atherosclerosis development [27].

## 5. Conclusions

A novel association between a functional polymorphism in *TLR4* (T399I) and COPD is reported. Considering that infections contribute to COPD and emphysema pathogenesis, our findings possibly indicate that dysfunctional polymorphisms of innate immune genes can affect the development of COPD in smokers. Although this finding warrants further investigation, it highlights the importance of impaired innate immunity towards COPD development.

## Figures and Tables

**Table 1:** Clinical and demographic data of study population.

Variable	COPD	Healthy
	patients	smokers
	(*n* = 134)	(*n* = 106)
Sex		
Male (*n*, %)	133 (99.25)	102 (96.23)
Female (*n*, %)	1 (0.75)	4 (3.77)
Age (years ± STDEV)	69.4 ± 8.9	63 ± 9.6
BMI (Kg/m^2^) (mean ± STDEV)	27.9 ± 5.8	27.7 ± 5.8
<30 Kg/m^2^ (*n*, %)	96 (71.6)	74 (69.8)
≥30 Kg/m^2^ (*n*, %)	38 (28.3)	32 (30.1)
Smoking status		
Current Smokers (*n*, %)	53 (39.5)	74 (69.8)
Ex Smokers (*n*, %)	81 (60.4)	32 (30.1)
Pys (mean ± STDEV)	70.9 ± 37.8	54.8 ± 23.3
COPD stage		
Mild (FEV_1_≥ 80%) (*n*, %)	6 (4.4)	
Moderate (FEV_1_: 50%–79%) (*n*, %)	74 (55.2)	
Severe (FEV_1_: 30%–49%) (*n*, %)	40 (29.8)	
Very severe (FEV_1_< 30%) (*n*, %)	14 (10.4)	
Frequency of exacerbations		
Frequent exacerbators (*n*, %)	34 (27.6)	
Nonfrequent exacerbators (*n*, %)	89 (72.3)	

Abbreviations: COPD, chronic obstructive pulmonary disease; BMI, body mass index; Pys, pack years.

**Table 2:** Distribution of the *TLR* SNPs in smokers with and without COPD.

Variable			COPD patients (*n* = 134)	Controls (*n* = 106)	*P*	OR (95% CI)
*TLR2*-R753Q	genotype	G/G (wt)	129	103	.311	
(2258G/A)		G/A (het)	5	3		
		A/A (hom)	0	0		
	alleles (*n*, %)	G	263 (98.1)	487 (96.7)	.317	0.54 (0.16–1.77)
		A	5 (1.9)	3 (3.3)		
*TLR4*-D299G	genotype	A/A (wt)	113	97	.095	
(896A/G)		A/G (het)	21	9		
		G/G (hom)	0	0		
	alleles (*n*, %)	A	247 (92.2)	203 (95.8)	.100	2.0 (0.87–4.57)
		G	21 (7.8)	9 (4.2)		
*TLR4*-T399I	genotype	C/C (wt)	112	98	**.039**	
(1196C/T)		C/T (het)	22	8		
		T/T (hom)	0	0		
	alleles (*n*, %)	C	246 (95.7)	204 (96.3)	**.044**	**2.4 (1.02**–**5.64)**
		A	22 (8.2)	8 (3.7)		
**Haplotypes **(*n*, %)						
TLR2-753R/TLR4-299D-399T - TLR2-753R/TLR4-299D-399T			110 (82.1)	90 (84.9)	.081	
TLR2-753R/TLR4-299D-399T - TLR2-753R/TLR4-299G-399I			20 (14.9)	8 (7.5)		
TLR2-753R/TLR4-299D-399T - TLR2-753R/TLR4-299G-399T			0 (0)	1 (0.9)		
TLR2-753R/TLR4-299D-399T - TLR2-753R/TLR4-299D-399T			1 (0.7)	0 (0)		
TLR2-753R/TLR4-299D-399T - TLR2-753Q/TLR4-299D-399I			2 (1.5)	7 (6.6)		
TLR2-753R/TLR4-299D-399T - TLR2-753Q/TLR4-299G-399I			1 (0.7)	0 (0)		

Abbreviations: COPD, chronic obstructive pulmonary disease; CI, confidence intervals; wt, wild-type; het, heterozygous; hom, homozygous.
